# Seasonal changes in predator community switch the direction of selection for prey defences

**DOI:** 10.1038/ncomms6016

**Published:** 2014-09-23

**Authors:** Johanna Mappes, Hanna Kokko, Katja Ojala, Leena Lindström

**Affiliations:** 1 Centre of Excellence in Biological Interactions Research, Department of Biological and Environmental Science, University of Jyväskylä, P.O. Box, 35, Jyväskylä FI- 40014, Finland; 2 Centre of Excellence in Biological Interactions Research, Department of Evolution, Ecology and Genetics, Research School of Biology, Australian National University, Australian Capital Territory 0200, Canberra, Australia

## Abstract

Insect communities consist of aposematic species with efficient warning colours against predation, as well as abundant examples of crypsis. To understand such coexistence, we here report results from a field experiment where relative survival of artificial larvae, varying in conspicuousness, was estimated in natural bird communities over an entire season. This takes advantage of natural variation in the proportion of naive predators: naivety peaks when young birds have just fledged. We show that the relative benefit of warning signals and crypsis changes accordingly. When naive birds are rare (early and late in the season), conspicuous warning signals improve survival, but conspicuousness becomes a disadvantage near the fledging time of birds. Such temporal structuring of predator–prey relationships facilitates the coexistence of diverse antipredatory strategies and helps explain two patterns we found in a 688-species community of Lepidoterans: larval warning signals remain rare and occur disproportionately often in seasons when predators are educated.

## Abstract (web summary)

The coexistence of alternative antipredatory strategies is poorly understood. Here, the authors show that warning colours lose their effectiveness when passerine birds, their main predators, fledge their young, which suggests that predators’ learning impacts selection for conspicuous warning signals.

## Graphical abstract

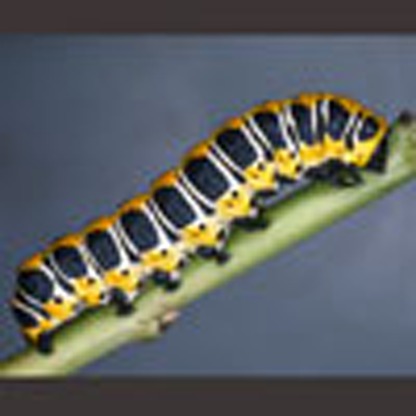

Animal colouration, ranging from camouflage to conspicuous warning signals, beautifully exemplifies the power of natural selection in action^1^,^2^. Camouflage evolved to conceal prey in the presence of predators, while conspicuous warning signals (aposematism) evolved to warn potential predators that a prey item is not profitable, that is, it has defences against predation^1^. Explaining the evolution of aposematism has been challenging^3^,^4^, as only most effective visual warning signals function as an anti-predatory strategy^2^,^5^,^6^ and the associated conspicuousness places a warning-coloured animal at risk^7^,^8^. To yield a net benefit, avoidance by educated predators must outweigh the mortality costs by naive predators^3^. However, unless avoidance has evolved to be fully innate, every predator generation has naive (young) predators^9^. If they target conspicuous prey and ignore the possible defence mechanism before the prey has been killed or injured, the problem of apparent altruism reappears continually: other prey individuals with similar phenotypes benefit as the predator becomes educated, but the individual educating the predator suffers a cost^4^,^5^. If the predators’ innate avoidance or neophobic behaviour is weak or absent^7^,^10^, explaining aposematism is challenging not only in its initial stages but in its maintenance phase too. The problematic need to educate naive predators never disappears^5^,^11^.

In seasonal environments, birds breed largely synchronously^12^. The age structure of the predator community therefore changes predictably over the breeding season^13^. The proportion of juveniles increases very rapidly as the progeny of the year start feeding independently. Juveniles have little experience of warning signals. If these naive predators have to learn to discriminate and avoid warning-coloured prey^5^, we can predict seasonal changes in the efficacy of warning signals, with the lowest advantage coinciding with the peak abundance of inexperienced predators. It is important to note that the advantages refer to relative survival. Absolute survival of prey may be additionally linked to variations in alternative prey availability^14^,^15^: in times of high insect abundance, per capita survival can increase despite breeding efforts of birds, also causing high predator activity. Although such factors have an impact on the absolute values of survival that can be expected, we focus here on changes in relative survival between prey types, as this determines the direction of selection (that is, whether warning colours or crypsis is favoured^16^,^17^).

To test for seasonal variation in the survival of cryptic or warning-coloured individuals created by a naturally occurring changes in the predator community^18^, we studied attack rates on three artificial^19^,^20^ larval types, which were either completely black (effectively cryptic; non-warning coloured) or had a small (moderately conspicuous) or a large (conspicuous) orange patch. We chose these three colour forms to investigate varying degrees of warning signal expression but also to match the natural variation in the warning signal expression of *Parasemia plantaginis*^21^. The black larvae are effectively cryptic as they blend into the shadows of natural vegetation. Our two choices for orange signals (small and large) resemble naturally occurring warning signals differing in conspicuousness and presumably the benefits in the presence of educated predators^22^. The large-scale field experiment was conducted in Central Finland, a highly seasonal environment, continually from spring (21 May) to fall (19 August), that is, before, during and after the nesting period of passerine birds (see Methods) in Finland.

We found that warning colours give relatively higher survival than crypsis early and late in the season. The survival of cryptic larvae peaks to exceed that of warningly coloured larvae in the middle of the season. These patterns of relative survival matches data on fledging times of passerine birds (main predators of larvae in vegetation), which suggests that juvenile fledging time increases the relative attack rate towards conspicuous larvae. Later in the season, the direction of selection switches back to favour of warning signals, which suggests a role for declining naivety in the predator community that has now undergone learning in the natural environment where non-artificial larvae combine warning colours with unpalatability. Crypsis yields similarly temporally structured survival advantages and disadvantages against both large and small warning signals. A separate experiment showed that warningly coloured prey is easier to detect from the vegetation than cryptic prey. Moreover, a seasonal pattern in warning colour occurrence was found among macrolepidopteran larvae, an important prey of most passerine birds breeding in the area^14^,^23^. Among 688 investigated lepidopteran larvae (all relevant species that exist in Finland), warning signals remain a rare strategy with <5% occurrence among all species as a larval colour pattern. They occur disproportionately often in seasons when adult birds dominate the predator community.

## Results

### Survival of artificial caterpillars in natural vegetation

**Figure 1:**
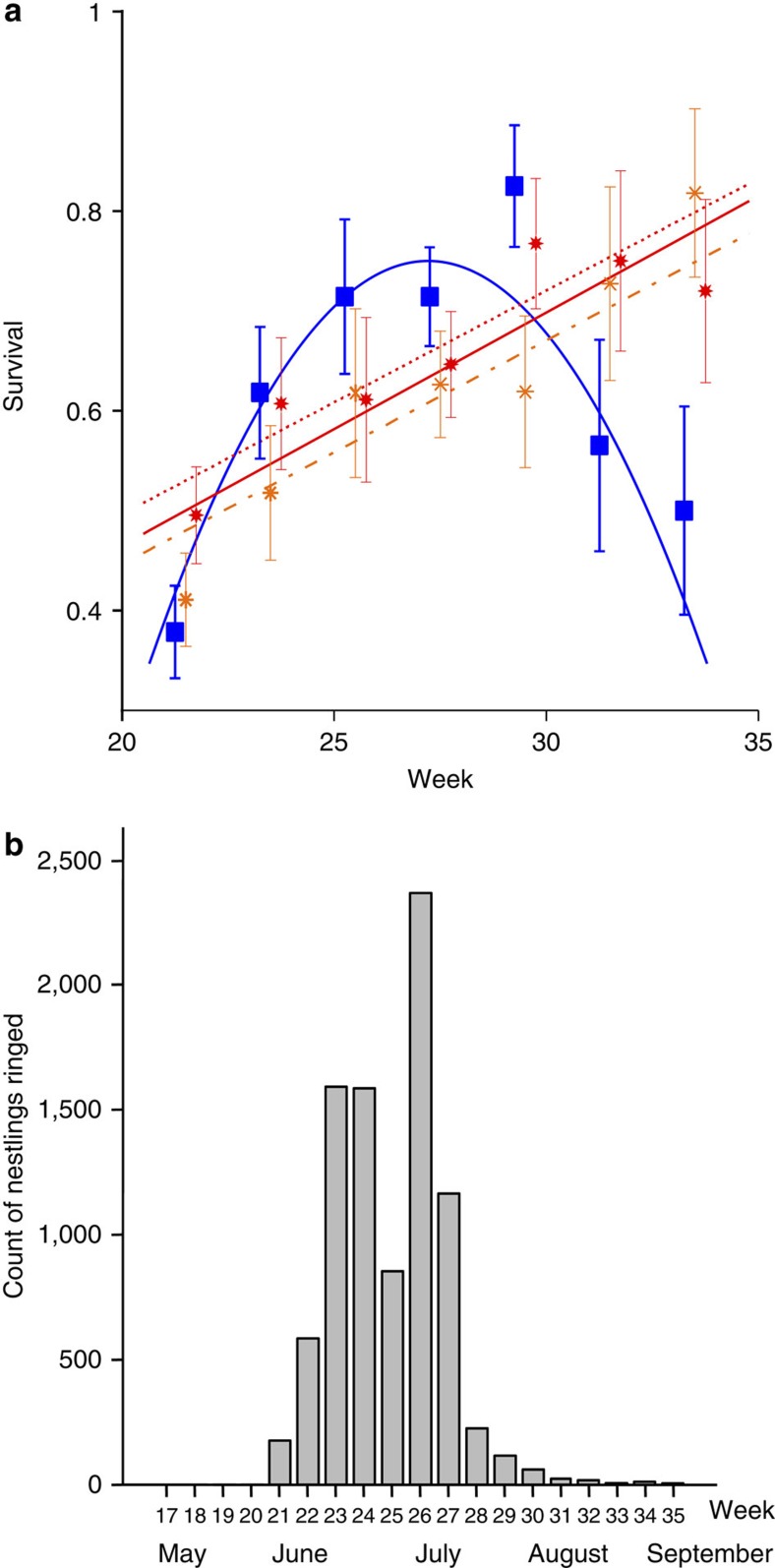
Seasonal changes of survival of larvae and emergence of juvenile birds. (**a**) Mean observed survival of three artificial larval types (*N*=1171, ±s.e.m.) over the season (non-warning-coloured: blue squares, small signals: orange stars, large signals: red bold stars) and the predictions for two best models (models D and E). The seasonal relationship for non-warning-coloured prey (blue curve) is identical in both models but differ for warning-coloured prey. Model D predicts an identical increase for small and large signals (red solid line) and model E a higher survival for large signals (red dotted line) than for small signals (orange line). (**b**) The estimated nest-leaving dates of juvenile passerine birds in Finland.

The survival of different prey types varied seasonally (Table 1 and Fig. 1a) with the pattern matching published data on fledging times of passerine birds (Fig. 1b), the main predators of larvae. We estimated seasonally varying survival differences among the three different larval types (cryptic, small warning signal and large warning signal) by fitting a set of candidate models to data and estimating their support based on Akaike information criteria (AIC)^24^,^25^ (Table 1). All models with at least moderate support, including the two models with clearly best support, agree on a general pattern in which the survival of warning-coloured prey increases over time and exceeds that of cryptic prey early and late in the season, but not in between (Table 1 and Fig. 1a). Thus, perfect and maximally strong signals are not required to yield the survival advantage once predators have started to avoid warning signals in general. The model selection procedure leaves it open whether large signals yield a consistent survival advantage over small signals (model E: small and large signal type have different intercepts) or not (model D: both warning-coloured prey types have identical survival that increases linearly over time, presumably because of increasing protection offered by growing vegetation and/or the availability of alternative prey). It is well known that a large, strong warning signal can promote predator-avoidance learning better^26^,^27^,^28^ compared with a small signal, but the relative benefit remains weak because of the detectability cost^17^,^27^.

### Changes in predator community structure

The changes in the survival of the artificial larvae match the temporal variation in the predator community structure, based on data of timing of young insectivorous birds leaving their nests and starting to feed on their own (Fig. 1b). The timing of naive predators in the predator community was calculated from the insectivorous birds’ nestling ringing data of 29 species (Supplementary Table 1), by estimating the timing of fledglings leaving their nests (approximately 1 week after ringing; The Finnish Ringing Centre). The same pattern can be found in a different data set of immature and mature birds (59 species, Supplementary Table 2) mist-netted throughout the summer (Supplementary Fig. 2). As the latter data set comprises birds that were caught in flight, it reflects the activity of the birds in each age group. From the nest-ringing data, the first passerine birds were estimated to leave their nests during week 21 (21–27 May), while the mist-netting data (Supplementary Fig. 2) shows that the first juvenile birds were caught 3 weeks later, during week 24 (11–17 June). The date when fledged birds start actively feeding on their own lies somewhere between these two estimates. Based on these two data sets, the peak date for the abundance of naive juveniles was estimated as 9 July (day 190 and week 28 from the beginning of the year; Fig. 1b).

### Warning colours on macrolepidopteran species

Given that the survival benefit of warning colouration changes seasonally and matches the temporal pattern in the experience level of insectivorous birds, we tested whether the consequences of temporally varying selection are visible in the prevalence of warning-coloured Lepidopteran larvae at different times of the breeding season of predators. Warning-coloured species should time their life cycle such that their larvae feed when the main fledgling period of predators is over and the juveniles have already gained experience with warning colours. If predators remember warning signals for a time period of several months^29^ or, in the case of migratory species, gain further education at overwintering sites, warning colouration keeps its benefits into spring, before the fledging of new juvenile predators.

**Figure 2:**
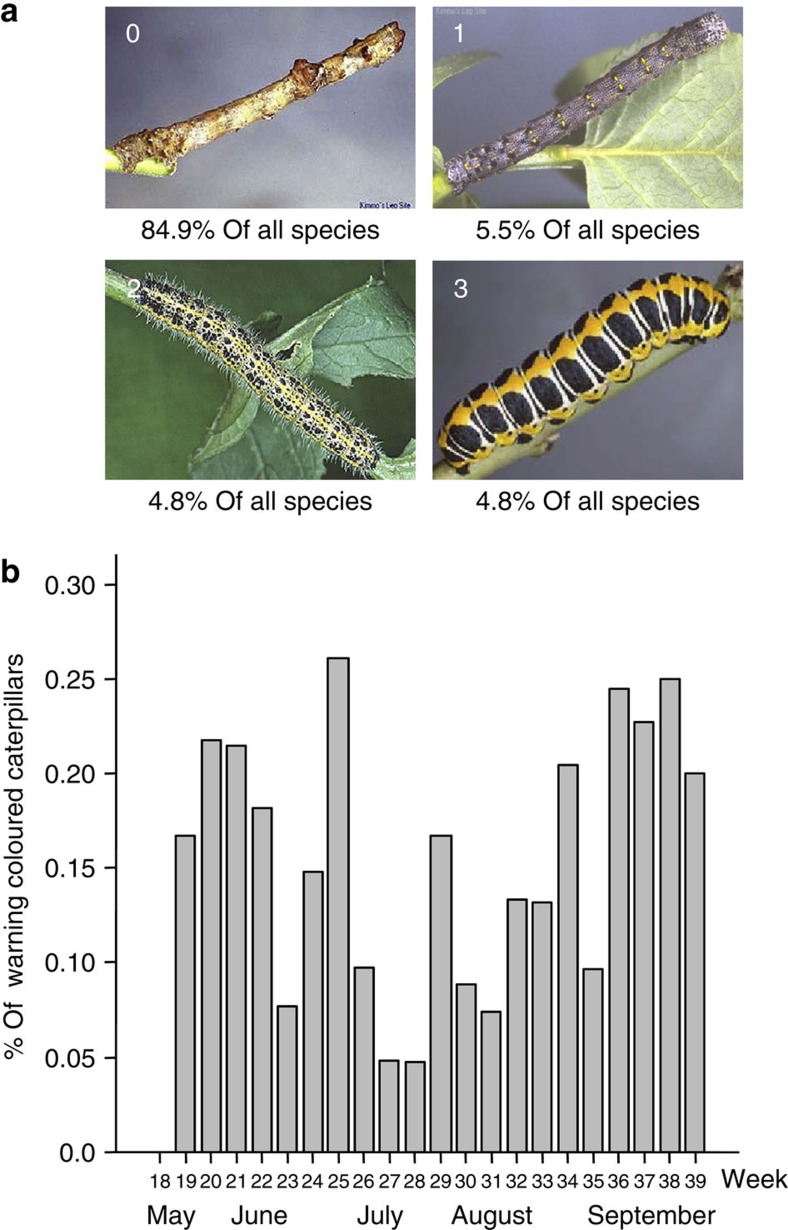
Examples of used categories of warning colouration of macrolepidopteran larvae and their seasonal occurrence. (**a**) Category 0, no typical warning colours (*Hypomecis roboraria*); category 1, some features of warning colouration (*Lycia lapponaria*); category 2, moderate warning colouration (*Pieris brassicae*); and category 3 (*Cucullia lactucae*), strong warning colouration. (**b**) Estimated proportion of warning-coloured larvae among 688 larvae (categories 1, 2 and 3) in the naturally occurring prey community. The estimation procedure uses abundance categories assuming that each abundance step corresponds to a fivefold increase in abundance, but assuming different step sizes than five reproduces the same pattern where signals are least prevalent in the middle of the season.

To examine the occurrence of free-living warning-coloured Lepidopteran species (we excluded those species, *N*=57, whose larvae live hidden in the stems of plants), we scored 688 photographs of Finnish macrolepidopteran larvae depicting warning colours of the last larval instar (see Methods). A total of 15.1% of larvae were classified as having features of warning colouration (classes 1–3, see Methods; see Fig. 2a for examples). Of these, 36.5% were classified as having some features of warning colouration, 31.7% as moderately warning coloured and 31.7% as strongly warning coloured. For each Lepidopteran species we also estimated the timing of occurrence and the abundance of last instar larvae (see Methods).

The prevalence of warningly coloured macrolepidopteran larvae was lowest during the main fledgling period of insectivorous birds, and the last-instar occurrence of a warningly coloured larva was significantly further away from the estimated peak juvenile time of insectivorous birds (9 July) than the corresponding timing of a non-warning-coloured larva (Fig. 2b and Supplementary Table 3a). This holds true both for spring (Supplementary Table 3b), when warning-coloured larvae occur earlier than non-warning-coloured ones, and for fall, where they occur later (Supplementary Table 3c). However, the relatively species-poor springtime data remain significant only in a part of the parameter range where we assume moderate to strong differences in larval abundance between species. As warning colouration is usually not only confined to the last instar of a larva, we checked whether our conclusions changed if the larval abundance was assumed to peak 10 days before the date given for the occurrence of the last instar; all significance levels remained unchanged.

## Discussion

Crypsis yields higher survival than warning colouration for artificial larvae as soon as fledglings begin to forage (Fig. 1a,b and Supplementary Fig. 2). Thus, naive predators can strongly and quickly alter selection for warning colouration versus crypsis. The relative advantage of crypsis declines rapidly: by the end of July, it has disappeared altogether. Our results highlight the ecological risks involved in warning colouration as a defence strategy against non-educated predators^11^,^30^ and suggest a clear reason for the relatively small number of Lepidopteran species (of 688 species, only 5% were classified as strongly signalling) that use this strategy. Certainly many biotic, abiotic and phylogenetic factors all affect larval colouration in Lepidopteran larvae, which can potentially restrain the evolution of warning colouration in many species. Nevertheless, as the small signalling artificial larvae survived equally well as the larvae with large warning signal (Fig. 1a), our results further suggests that conspicuousness against predators is costly^31^. A large orange patch on the black larva makes it an easy target to detect^22^, which makes such lifestyle dangerous whenever there is a risk that environment features naive predators^17^.

Although our focus was on the direction of relative survival, which switches twice, it also appears appropriate to comment on overall trends in absolute survival. The abundance of alternative prey^14^ increases over the summer, as does the height and density of the vegetation; such factors are potential candidates for explaining why Fig. 1a as a whole features increasing survival (per prey item) over the season. As our data set relies on naturally occurring temporal variation, we have not removed the fact that several seasonal changes co-occur. Teasing apart the role of each explanation is consequently not simple; for example, the conspicuousness difference between warningly coloured prey and cryptic prey could increase as the vegetation grows more dense. However, it is noteworthy that any explanation that relies on a variable that increases (or decreases) continually over the season is an unlikely alternative explanation for our observed pattern where crypsis is favoured both early and late in the season; our preferred explanation, the naivety of the predator community, offers a clear temporal peak matching our results, as well as a readily applicable mechanism, as predator learning is already known to have an impact on prey choice^9^,^31^,^32^.

As our field experiment did not allow repeated exposure to artificial larvae, any learning must have happened through encounters with naturally occurring prey. Our results thus give further evidence for the importance of the fact that predators generalize among warning signals (see refs 33, 34, 35 for evidence that generalization can be wide). Generalized learning explains why aposematic organisms can trust their warning colours as soon as most predators have learned to avoid them. Yet, in the middle of every summer, the problems related to the initial evolution of aposematism recur: there is a continuous need to educate a large number of naive predators^5^. Accordingly, warning colours are less often found in larvae that occur during this time of the year. The fast decline of the benefit of crypsis implies, however, that young predators learn sufficiently fast so that late-occurring larvae enjoy an environment in which predators are already educated. The seasonal changes in the colouration of the real larval community suggest that this is evolutionary significant: later occurring species essentially exploit the education effort by earlier species. Given that there are other factors that can constrain the timing of life history events (direct effects of weather on growth^15^,^36^ and metabolic rate^37^, and the seasonal availability^38^ or quality^39^,^40^,^41^ of food plants), our work uncovers an intriguing possibility of a multi-species evolutionary game where it is safer to feed later in the season than other prey, but a combination of other fitness components prevent all species from exploiting the late niche of relative safety equally. This gradual change from crypsis-dominated prey community to one displaying more warning colours, in turn, provides predators with opportunities to learn over time, a requisite for maintaining the pattern that we observe.

## Methods

### Survival of artificial caterpillars in natural vegetation

To estimate the efficacy of protective uation in different seasons with changing predator community age structure, we conducted experiments using three types of artificial plasticine larvae (length: 3.85 cm; width: 8 mm, Black: Caran d’Ache Modela colour 0259.009 and orange: Creative Art Modeling clay) with either a small (round: a girth of 4–5 mm) or large (patch length: 3.45 cm; width: 7 mm) orange warning signal located roughly one quarter from one end of the larva, or no warning colours at all (larvae all black) (Supplementary Fig. 1). The total number of artificial larvae was 1,243. We estimated the attack rate by wild birds on these larval types by attaching larvae triplets with a metal wire to natural vegetation (at height 20–75 cm) in open and semi-open environments in the Jyväskylä area of southern Finland (62°14′ N, 24° 43′ E). All three different types of larvae were placed in all types of backgrounds. To mimic the natural conditions, larvae were placed on the stems and lower parts of plants rather than on the leaves and upper part of plants where they would maximize their visibility to predators. Larvae were distributed widely (over an area of about 50 km^2^). Different triplets were never closer than 20 meters apart and larvae within the triplet were placed 5 m apart. Experimental days, which occupied 19 different dates spanning 21 May (week 21) to 19 August (week 33)—with at least one experimental date per week, except week 25—involved placing an average of 20 triplets; the same location was never used twice; therefore, any single predator was unlikely to re-encounter artificial larvae during the experiment. The larvae were checked for bird beak marks 5 days after they were attached to vegetation. Beak-marked larvae were recorded as killed. Excluding the 72 larvae lost with an unknown fate yields data on 1,171 larvae in total.

To ensure that all three larval types were positioned equally, we assessed the visibility (0=not visible, 1=partly visible and 2=totally visible) of the larvae from above, as well as from 2 m distance horizontally from four orthogonal directions. There was no difference in the visibility of the different larval types (one-way analysis of variance F(2,1165)=0.111, *P*=0.985).

### Signal conspicuousness

To test the visibility of the three types of signals, we attached 30 artificial larvae, 10 of each signal type (black, small orange signal and large orange signal), to natural vegetation with a metal wire at height of 30–90 cm. The area was 20 m × 20 m and the vegetation height was about 120 cm. The dominant plants were tea-leaved willows (*Salix phylicifolia*) and fireweeds (*Epilobium angustifolium*). Larvae were placed randomly in the vegetation. Twenty-one (*n*=21) biology students, who had been shown the three types of larvae before the experiment, each searched for the larvae for 7 min and the order in which they found different larval types was recorded. Each different larvae types found were given a score: first 1, second 2 and so on; 384 out of 600 larvae were found by the students. Next, we calculated the total scores for each larval type; low scores indicate high conspicuousness. The larvae with a large orange signal (score mean=154.5, s.e.m.=5.8) were the easiest to find (Wilcoxon signed-rank test, Bonferroni-corrected black versus large orange signal *Z*=−2.573, *P*=0.030; small versus large orange signal *Z*=−3.546, *P*<0.001). There was no difference in conspicuousness between a small orange signal (score mean =204.5, s.e.m.=6.1) and the black larvae (score mean=185.7, s.e.m.=7.7; Wilcoxon signed-rank test black versus small orange signal *Z*=−1.999, *P*=0.138). The fact that large warning signals carry detectability costs is in line with our other findings concerning bird predators^22^.

### Estimation of the survival of artificial larvae

Complying with statistical recommendations, we kept the set of candidate models small^24^,^25^ (Table 1). Each model specifies the 5-day survival probability *S*_5_ as either a linear function of time, *S*_5_(*d*)=*a*+*bd* (*d* denoting days since 1 January), or a quadratic form *S*_5_(*d*)=*a*+*b*(*d*−*c*)^2^ that peaks at day *d*=*c*. The model does not force the peak to occur within the time frame of the experiment; thus, the quadratic form also allows for curved shapes with, for example, continually improving survival throughout the experiment. Models vary in the number of parameters, depending on whether the model is linear or quadratic, whether different larval types had independent or identical parameter values and whether the position of the peak was set *a priori* or not. Thus, we tested alternatives ranging from model A, which has no differences between larval types and assumes a linear relationship with time for all of them, to model H, where each larval type follows its own non-linear pattern with time. AIC scores were calculated based on the parameter values that maximized the likelihood of observing the pattern of binary death or survival, 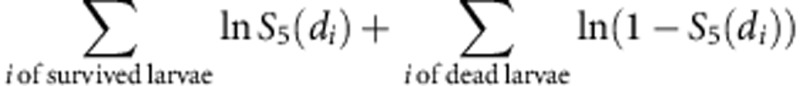, where *d*_*i*_ is the day of placing the larva in vegetation. As an illustrative example of the use of this modelling approach, if a linear model uses parameters *a*=0.1 and *b*=0.001, then *a*+*bd*_*i*_ on, for example, day *d*_*i*_=180 would predict *S*_5_(*d*_*i*_)=0.28. If we then observed that 5 out of 15 larvae placed that day survived, the log likelihood of this observation is obtained as the above sum and equals 5 ln(0.28)+10 ln(1−0.28)=−9.65. If another model produces *S*_5_(*d*_*i*_) that is closer to the observed survival (5/15 in this example), then its log likelihood is higher. The entire data set over the entire season provides the sum of all such values, with the predictions *S*_5_(*d*_*i*_) obviously changing with *d*_*i*_ as the season progressed. The AIC approach enters the best total log-likelihood obtainable within each model structure to form a tabulated list of the AIC values, which additionally penalize models that include many parameters^18^,^19^.

Note that the AIC differences produced are identical to an approach that considers the likelihood of observing a specific number of surviving larvae based on the binomial distribution grouped according to weeks (the equivalence arises because the factorials of each binomial distribution are necessarily identical across models for the same data sets).

### Predator community structure

We estimated temporal changes in predator community based on two different data sets. The ringing data (obtained from the Finnish Ringing Centre) comprises 29 insectivorous passerine species ringed in southern Finland in the summer of 2005, total *n*=8,797. It reflects the onset of the predictable change in the predator community, allowing us to estimate when naive predators start feeding on their own. The mist-net data set (likewise obtained from the Finnish Ringing Centre) gives us an estimate of when the juveniles are abundantly foraging on their own.

Note that birds that nest in nest boxes are overrepresented in the ringing data due to the relative ease of finding their nests. In addition, some very common species may be underrepresented if they fail to attract the interest of ringers in proportion to their abundance. Yet, we do not believe such issues to cause a bias in assessing the nest-leaving date: although the peak laying date of species breeding in nest boxes is later than those with open nests, the earliest chicks fledge at the same time.

The mean age of ringed chicks was 7 days and passerine birds usually leave their nest at the age of 14 days (The Finnish Ringing Centre). Thus, we estimated the nest-leaving date by adding 7 days to the date of the ringing of the chicks. However, fledglings do not begin feeding on their own immediately after leaving their nest; they are mainly dependent on their parents’ feeding for at least a week (The Finnish Ringing Centre). Therefore, we expect the young birds to begin to have a significant effect on the prey species on average 2 weeks after ringing. We additionally estimated the changes in insectivorous bird community structure based on a data set of mist-netted birds (59 species) in 14 localities (total *n*=3,925, comprising 1,666 adult and 2,259 immature birds) throughout the summer 2005 in southern Finland. The mist nettings were performed between 1 May and 3 September, 12 times in each locality, but on different dates in different localities. The birds born in the summer 2005 were recorded as immature (juvenile) and the individuals from previous years were recorded as adults (ad). As this data is based on birds that were caught in flight, it tells us about the activity of the birds in each age group (see Supplementary Fig. 2). By comparing this data with the nest ringing data, we obtain a more complete estimate of the time when juvenile birds start feeding actively and their proportion in the predator community.

### Warning colours on macrolepidopteran species

Southern Finland (below latitude 66°) has 759 resident macrolepidopteran species, of which we scored 750 for warning colours on the last larval instar. Scoring was performed based on slides taken by Kimmo Silvennoinen, who has hand reared the larvae and photographed each specimen on their natural host plants. The non-scored nine species were the rarest ones and thus unlikely to affect our analysis. From the final analysis (Supplementary Table 3), we excluded those species (*n*=57) that complete their larval life within the host plant (for example, *Ipimorpha retusa*) and are thus not exposed to visually hunting predators. The final analysis thus comprised 688 Lepidoteran species.

Two evaluators, who are keen lepidopterists (KO and KS) assigned species to one of four categories (see Fig. 2a):

1. no typical warning colours: typical warning colours (for example, yellow, red, orange combined with black) not present. Colourful larvae that match their host plant colouration as well as the masquerading caterpillars were included in this category (*n*=584).
2. some features of warning colouration: small, specific typical warning colours such as orange, yellow or red areas on otherwise relatively cryptic body (*n*=38). Species that had small coloured patterns positioned in such a way that it made the larva cryptic or the larva that had colouration matching their background were assigned to category 0.
3. moderate warning colouration: clear orange, yellow or red areas, or conspicuous black and white patterns (*n*=33).
4. strong warning colouration: extremely conspicuous; large orange, yellow or red areas, often combined with black or white (*n*=33).

When the evaluators did not agree in their assessment (3.9% of the species), further pictures of larvae in their natural environment were checked to reach agreement. Some of the species are polymorphic in their appearance; in these cases we classified the species according to the more numerous morph. Note that as we only examined whether the species had typical warning colours against their natural host plant, it is possible that some species with atypical warning signals (for example, ultraviolet colours^42^) were scored as non-warning coloured. Ultraviolet signals are, however, known to be extremely rare in caterpillars^43^. Moreover, it is noteworthy that our classification does not distinguish between aposematic organisms and their Batesian mimics, as we did not study whether each species has chemical defence against predation.However, it is also noteworthy that the temporal switch in selection is expected to apply to both categories equally.

We recognize that our classification to four categories was crude, as bird vision is known to differ from the human vision. For instance patterning, in addition to colours, can also influence detectability: for example, elongated patterns are often more visible than small dots^2^. However, elongated pattern elements typically are also larger in size and will be classified more conspicuous than a specimen with the same colours arranged in small dots. We aimed at a scale of analysis that prevents us from acquiring fresh samples of all caterpillar larvae required by standard colour analysis, and available data on lepidopteran colours and their bird-eye-corrected conspicuousness on their natural backgrounds^44^,^45^ match our classification. All such analyses agree that lepidopteran pigments such as bright yellow, orange and red are conspicuous on all natural backgrounds (typically leaves or tree trunks), whereas green, brown and grey seldom are overtly conspicuous on vegetation.

For each species, we also estimated the time when each of the species occurs as a last-instar larvae in southern Finland^46^,^47^,^48^,^49^,^50^,^51^. The estimation of the timing of the large larvae of a species was based on its life cycle. We recorded both the timing of the adult flight of adults as well as the overwintering stage of the larvae. The last-instar larvae were estimated to occur 2 weeks before the onset of the adult flight. If the species had more than one generation per year (*n*=32, 4.7% of the species), we used the phenology of the first generation, as it is usually the more numerous one. Based on literature^46^,^47^,^48^,^49^,^50^,^51^, we also scored the abundance of the species based on adult abundance. The abundance of each species was categorical from 1 (extremely rare; found only one at the time) to 10 (extremely abundant; usually species dominating the community). We analysed the data assuming that each category step corresponds to an *k*-fold increase in abundance, such that the nine abundance steps between the least abundant category 1 and most abundant category 10 reflect an abundance difference of *k*^9^. The true value of *k* is unknown, but when the same pattern of time dependence of the prey community structure is obtained using *k*-values ranging from almost certain underestimates to equally certain overestimates, the results can be considered robust.

## Author contributions

J.M., L.L. and K.O. designed the experiment; K.O and J.M. performed the field experiment. K.O. and K.S. scored the Lepidopteran larvae, and H.K. performed the AIC analysis. All authors contributed to the writing of the manuscript.

## Additional information

**How to cite this article:** Mappes, J. *et al.* Seasonal changes in predator community switch the direction of selection for prey defences. *Nat. Commun.* 5:5016 doi: 10.1038/ncomms6016 (2014).

## Supplementary Material

Supplementary Information Supplementary Figures 1-2 and Supplementary Tables 1-3

## Figures and Tables

**Table 1:** The eight tested models, model outcome in terms of the temporal pattern of survival differences between larval types.

Model (*i*)*	AIC	Δ_*i*_	*W*_*i*_	Temporal pattern†
D warning-coloured: identical linear cryptic: non-linear	1,449.21	0	0.36	Both signals: W (5 June) C (27 July) W
E warning-coloured: linear diff.intercept cryptic: non-linear	1,449.28	0.07	0.35	Small signal: W (3 June) C (30 July) WLarge signal: W (8 June) C (24 July) W
F warning-coloured: different linear cryptic: non-linear	1,450.09	0.88	0.23	Small signal: W (31 July) C (28 July) WLarge signal: W (10 June) C (26 July) W
H all different, non-linear	1,452.76	3.55	0.06	Small signal: W (28 May) C (24 July) WLarge signal: W (7 June) C (22 July) W
G all identical, non-linear	1,459.91	10.70	<0.01	No difference between larval types
A all identical, linear	1,466.85	17.64	<0.01	Survival of all types increases with time
B all linear, warning-c diff. from cryptic	1,470.23	21.02	<0.01	Survival of all types increases with time
C all different and linear	1,471.33	22.12	<0.01	Survival of all types increases with time

AIC, Akaike Information Criteria.

Models are listed in order of increasing AIC values (AIC, score differences Δi and Akaike weights Wi).

^*^The model description uses ‘identical’ to denote that parameter values were shared between larval types, and ‘different’ to denote that parameters were estimated separately. ‘Nonlinear’ refers to a quadratic form (survival=a+bd+c2), where d=date (from the beginning of the year), and a, b and c are parameters. ‘Linear’ refers to survival=a+bd, and ‘diff.intercept’ to a linear model in which two larval types share the same slope b but the intercept a is estimated separately for each type.

^†^The column ‘temporal pattern’ summarizes the outcome by giving the date-specific winning type. Thus ‘W (5 June) C (27 July) W’ means that the model predicts the warning-coloured type to have higher survival than the cryptic type before 5 June and after 27 July but not between those dates. This is given separately for the different warning-colour signals where the model differentiates between them.
