# Supplementary material for: Seasonal changes in predator community switch the direction of selection for prey defences
Source: Nat Commun. 2014 Sep 23;5:5016. doi: 10.1038/ncomms6016 (PMC4199109; doi:10.1038/ncomms6016)
Supplement: Supplementary Information — Supplementary Figures 1-2 and Supplementary Tables 1-3 [file ncomms6016-s1.pdf]

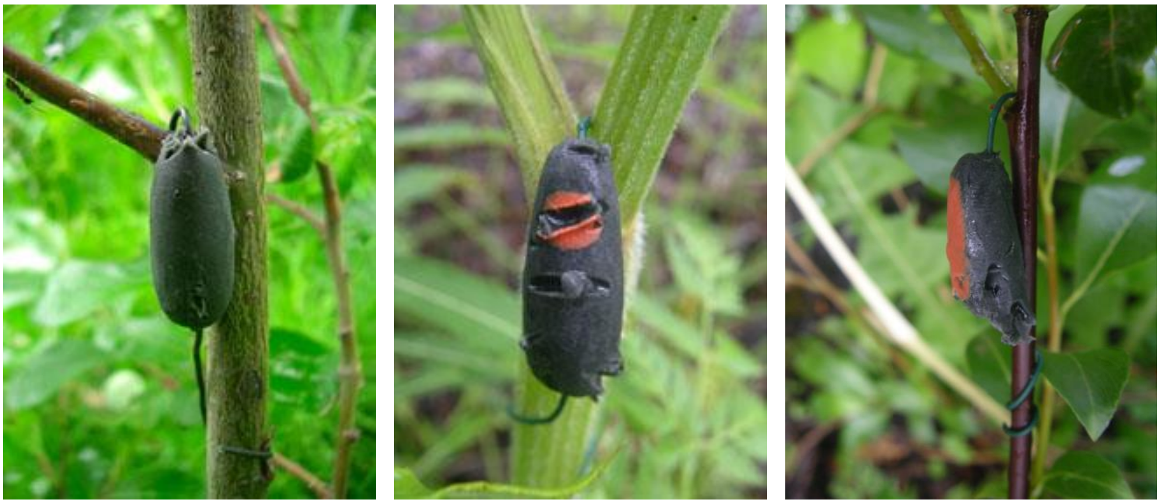

Supplementary Figure 1. Three attacked artificial plasticine larvae in the experiment. We used three color forms of artificial plasticine larvae which were either completely black (effectively cryptic; non-warning coloured) or had either a small (moderately conspicuous) or large (conspicuous) orange patch in the experiment. This figure illustrates how the plasticine artificial larvae store the attacks by the predators. Note that these pictures are chosen on the basis of the attacks.

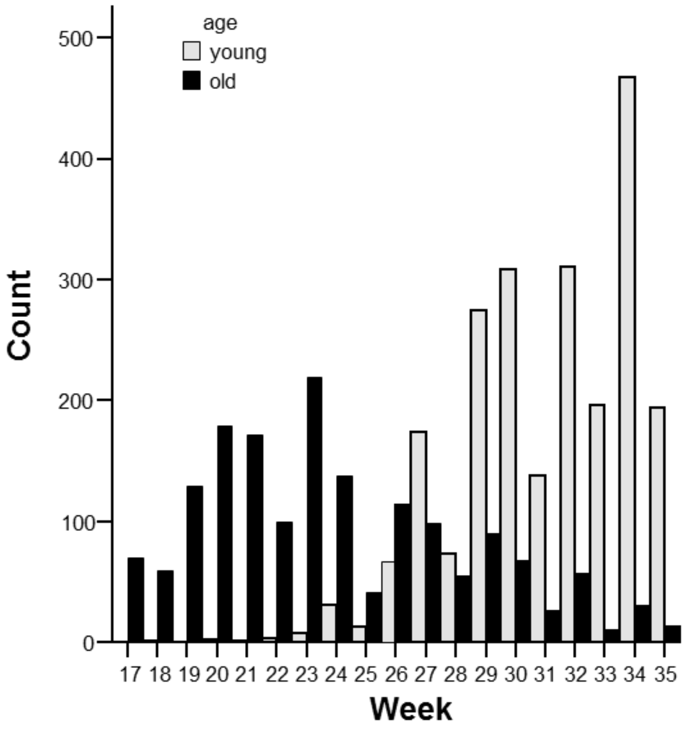

Supplementary Figure 2. The number of mist-netted young and adult birds during summer 2005. The mist-net data clearly shows that the young start to move around the week 25. Week 17 corresponds to (First week of May) and week 35 to (Last week of August)

Supplementary Table 1. List of species ringed as fledglings. We used data on 29 species of insectivorous birds ringed as nestlings in continental southern Finland in the summer of 2005. Data was provided by the Finnish Ringing Agency, University of Helsinki.

Bird species:	Frequency	Percent
Anthus pratensis	8	0.09
Carduelis cannabina	10	0.11
Carduelis chloris	23	0.26
Carpodacus erythrinus	5	0.06
Emberiza citrinella	11	0.13
Emberiza schoeniclus	25	0.28
Erithacus rubecula	41	0.47
Ficedula hypoleuca	3255	37.00
Fringilla coelebs	35	0.40
Motacilla alba	100	1.14
Motacilla flava	115	1.31
Muscicapa striata	183	2.08
Oenanthe oenanthe	10	0.11
Parus ater	240	2.73
Parus caeruleus	915	10.40
Parus cristatus	127	1.44
Parus major	1278	14.53
Parus montanus	31	0.35
Phoenicurus phoenicurus	203	2.31
Phylloscopus trochilus	98	1.11
Phylloscopus sibilatrix	27	0.31
Prunella modularis	33	0.38
Saxicola rubetra	13	0.15
Sylvia atricapilla	5	0.06
Turdus iliacus	971	11.04
Turdus merula	49	0.56
Turdus philomelos	113	1.28
Turdus pilaris	864	9.82
Turdus viscivorus	9	0.10
Total individuals	8797	100.00

Supplementary Table 2. Frequencies of young and adult birds. Data comprises the mist-nettings of 59 insectivorous birds ringed in southern Finland in the summer of 2005. Data was provided by the Finnish Ringing Centre, University of Helsinki.

	Frequency		Total	Percent
Bird species	young	adult		%
Acrocephalus dumetorum	1	2	3	0.0008
Acrocephalus palustris	4	8	12	0.0031
Acrocephalus schoenobaenus	271	217	488	0.1243
Acrocephalus scirpaceus	22	17	39	0.0099
Aegithalos caudatus	2	0	2	0.0005
Alauda arvensis	0	1	1	0.0003
Anthus pratensis	0	1	1	0.0003
Anthus pratensis	11	7	18	0.0046
Carduelis cannabina	1	0	1	0.0003
Carduelis carduelis	3	0	3	0.0008
Carduelis chloris	80	36	116	0.0296
Carpodacus erythrinus	29	63	92	0.0234
Carduelis flammea	53	22	75	0.0191
Carduelis spinus	43	31	74	0.0189
Certhia familiaris	8	1	9	0.0023
Dendrocopos major	0	2	2	0.0005
Dendrocopos minor	0	4	4	0.0010
Emberiza citrinella	4	23	27	0.0069
Emberiza pusilla	0	1	1	0.0003
Emberiza schoeniclus	68	94	162	0.0413
Erithacus rubecula	384	68	452	0.1152
Ficedula hypoleuca	61	71	132	0.0336
Ficedula parva	2	2	4	0.0010
Fringilla coelebs	22	92	114	0.0290
Fringilla montifringilla	2	0	2	0.0005
Garrulus glandarius	0	3	3	0.0008
Hippolais icterina	5	4	9	0.0023
Jynx torquilla	1	5	6	0.0015
Lanius collurio	12	17	29	0.0074
Locustella fluviatilis	0	1	1	0.0003
Locustella naevia	5	3	8	0.0020
Luscinia luscinia	14	20	34	0.0087
Luscinia svecica	0	5	5	0.0013
Motacilla alba	12	9	21	0.0054
Muscicapa striata	15	18	33	0.0084
Oenanthe oenanthe	0	1	1	0.0003
Parus caeruleus	103	34	137	0.0349
Parus cristatus	3	0	3	0.0008
Parus major	117	76	193	0.0492
Parus montanus	21	7	28	0.0071
Phoenicurus phoenicurus	16	8	24	0.0061
Phylloscopus collybita	15	13	28	0.0071

Phylloscopus trochilus	404	286	690	0.1758
Phylloscopus sibilatrix	5	2	7	0.0018
Prunella modularis	25	14	39	0.0099
Pyrrhula pyrrhula	4	4	8	0.0020
Regulus regulus	1	3	4	0.0010
Saxicola rubetra	1	4	5	0.0013
Sturnus vulgaris	4	3	7	0.0018
Sylvia atricapilla	46	17	63	0.0161
Sylvia borin	111	107	218	0.0555
Sylvia communis	63	52	115	0.0293
Sylvia curruca	84	30	114	0.0290
Sylvia nisoria	1	1	2	0.0005
Troglodytes troglodytes	4	0	4	0.0010
Turdus iliacus	32	62	94	0.0239
Turdus merula	29	28	57	0.0145
Turdus philomelos	17	25	42	0.0107
Turdus pilaris	18	41	59	0.0150
	2259	1666	3925	

Supplementary Table 3. Differences in timing of the occurrence between the larvae with or without warningly colors.

(a) The weighted mean difference between the timing of a last instar larvae (*T*) and 9th of July (day 190 of the year), weighted with larval abundance:

$\sum_{\text{species}} c^k |T - 190| / \sum_{\text{species}} c^k$, where *c* is the abundance category of a species and values of *k* are varied to perform sensitivity analysis. Statistical significance tests were performed by comparing the observed difference between weighted means of warning-colored and non-warning-colored larvae to a distribution obtained by randomly permutating the data of warning coloration (present or not) 10000 times, and scoring the difference of weighted means for each permutation. (b) and (c) as in (a), but analyzed separately for species that occur in the spring to early summer (that have $T \leq 190$) and late summer to autumn ($T > 190$), where weighted mean dates are now given rather than the difference to day 190.

(a) Absolute difference: warning-colored larvae have greater mean distance (in days) from day 190

	Warning colors		P
	present (n=104)	not present (n=584)	
(i) *k* = 3	53.65	32.92	0.0029
(ii) *k* = 5	60.06	30.83	0.0023
(iii) *k* = 10	67.65	29.28	0.0018

(b) Springtime occurrence: warning-colored larvae occur earlier

	Warning colors		P
	present	not present	
	(n=19)	(n=120)	
(i) *k* = 3	176.07	181.72	0.061
(ii) *k* = 5	174.98	182.44	0.043
(iii) *k* = 10	173.44	182.83	0.029

(c) Autumn occurrence: warning-colored larvae occur later

	Warning colors		P
	present	not present	
	(n=85)	(n=464)	
(i) *k* = 3	249.13	231.59	0.017
(ii) *k* = 5	254.12	229.85	0.016
(iii) *k* = 10	260.08	228.71	0.015
